# *Tetrahymena* predation drives adaptive evolution of *Salmonella* by disrupting O-antigen biosynthesis and upregulating transcriptional regulator *csgD*

**DOI:** 10.1093/ismejo/wraf070

**Published:** 2025-04-14

**Authors:** Hao Huang, Jinzhu Geng, Yuhao Dong, Chen Yuan, Gang Li, Meng Nie, Jingjing Guo, Yongjie Liu

**Affiliations:** Joint International Research Laboratory of Animal Health and Food Safety, College of Veterinary Medicine, Nanjing Agricultural University, Nanjing 210095, Jiangsu, China; Joint International Research Laboratory of Animal Health and Food Safety, College of Veterinary Medicine, Nanjing Agricultural University, Nanjing 210095, Jiangsu, China; Joint International Research Laboratory of Animal Health and Food Safety, College of Veterinary Medicine, Nanjing Agricultural University, Nanjing 210095, Jiangsu, China; Joint International Research Laboratory of Animal Health and Food Safety, College of Veterinary Medicine, Nanjing Agricultural University, Nanjing 210095, Jiangsu, China; Joint International Research Laboratory of Animal Health and Food Safety, College of Veterinary Medicine, Nanjing Agricultural University, Nanjing 210095, Jiangsu, China; Joint International Research Laboratory of Animal Health and Food Safety, College of Veterinary Medicine, Nanjing Agricultural University, Nanjing 210095, Jiangsu, China; Joint International Research Laboratory of Animal Health and Food Safety, College of Veterinary Medicine, Nanjing Agricultural University, Nanjing 210095, Jiangsu, China; Joint International Research Laboratory of Animal Health and Food Safety, College of Veterinary Medicine, Nanjing Agricultural University, Nanjing 210095, Jiangsu, China

**Keywords:** protozoan predation, adaptive evolution, Salmonella, genetic mutation, transcriptional regulation

## Abstract

Protozoan predation is increasingly understood to be one of the main environmental factors driving bacterial virulence evolution and adaptation strategies. In this study, we reveal the adaptive evolution of *Salmonella* Enteritidis in phenotypic and genomic traits after passage through *Tetrahymena thermophila*. We identified a beneficial and fixed mutation that occurs at the coding region of *rfbP*, encoding the undecaprenyl-phosphate galactose phosphotransferase, and demonstrated that almost all observed phenotypic changes caused by selection pressure, including enhanced biofilm formation and reduced bacterial motility, are related to the early termination of RfbP protein translation. This mutation blocks the lipopolysaccharide O-antigen synthesis and leads to upregulation of the transcriptional factor *csgD*, which plays a central role in regulating *Salmonella* adaptation to the adverse environment. Our findings underscore the selective pressure from *Tetrahymena* as a pivotal driver of adaptive evolution in *Salmonella*, elucidating the nexus between adaptation to protozoan predation and augmented environmental persistence. This investigation advances our understanding of the ecological role of protozoan predation in the natural selection of bacterial communities.

## Introduction

*Salmonella* is an environmentally ubiquitous Gram-negative bacterium, with >2500 different serotypes. *Salmonella* Enteritidis, one of the most common types, is a zoonotic foodborne pathogen of worldwide importance, which not only causes significant economic losses in the animal industry but also poses a serious threat to human health [1]. Previous studies have documented the ability of this bacterium to survive for extended periods of time within secondary habitats including water [2, 3].

In microbial ecosystems, the interactions between protozoans and bacteria extend beyond the simple predator–prey relationship [4, 5]. This dynamic interaction, especially protozoan predation, imposes a natural selective pressure on bacteria, affecting their environmental adaptability and promoting their persistence within hosts [6, 7]. Protozoan predation represents one of the environmental challenges faced by bacteria, acting as a catalyst for bacterial evolution and adaptive enhancement [8]. It has been reported that *Acanthamoeba* and *Tetrahymena*, upon ingesting *Legionella pneumophila* [9–11], *Escherichia coli* [12], *Listeria monocytogenes* [13, 14], *Aeromonas hydrophila* [15], and *Stenotrophomonas maltophilia* [16], could release expelled food vacuoles (EFVs) containing viable bacterial cells. Specifically, bacteria within EFVs exhibit increased resistance to acidic conditions, freeze–thaw cycles, ultrasonication, and exposure in cooling tower bioreactors [17]. Under starvation conditions, cells within EFVs show enhanced survival rates, potentially lasting at least 6 months [11]. This resistance to stress and prolonged starvation may facilitate absorption and infection by hosts. In *Vibrio cholerae*, adaptations to the amoeba host lead to reduced motility, biofilm formation, and hemolytic activity, along with increased protease activity compared to non-adapted strains [18]. *V. cholerae* EFVs survive better than planktonic free-living cells under different stresses and show an increased infectivity [19]. These changes likely represent adaptive responses to protozoan predation pressure.

Our current understanding of the selective grazing effects of bacterivorous predation on bacterial survivability and adaptability is limited, as most studies have primarily focused on investigating the phenotypic alteration rather than on the reason behind it. This is likely due to the variety of protozoa displaying different mechanisms. In *V. cholerae*, evolved adaptive traits in response to amoeba predatory pressure were demonstrated to be linked to a nonsynonymous mutation in the flagellar transcriptional regulator *flrA* [18]. In *A. hydrophila*, phage resistance caused by *Tetrahymena thermophila* predation was due to the downregulation of a flagellar biosynthesis regulator, *flhF*, by enhancing secretion of extracellular protein that hinders phage adsorption [20]. One previous study indicated that protozoan predation exerts selective pressure on *S.* Enteritidis, enhancing its environmental adaptability and intracellular persistence, thereby facilitating its natural evolution [21]. However, the factors driving these phenotypic changes are unknown. This study is the first to reveal rapid adaptive evolution of *S.* Enteritidis under predation pressure imposed by *T. thermophila* through causative genetic mutations and gene regulatory mechanisms. These findings contribute significantly to our knowledge of how microbes persist in the environment.

## Materials and methods

### Strains, plasmids, and culture conditions

Bacterial strains, plasmids, and primers used in this study are listed in Table S1. All bacterial strains were routinely grown at 37°C in Luria Bertani (LB) medium (Oxoid, UK) under standard laboratory conditions. *T. thermophila* SB210 [22], obtained from Dr. Miao Wei, Institute of Hydrobiology, Chinese Academy of Sciences, was grown axenically in SPP medium (containing 2% proteose peptone, 0.1% yeast extract, 0.2% glucose, and 0.003% EDTA-Fe) at 28°C. When required, the final concentrations of antibiotics in growth media were as follows: 100 μg/ml of gentamicin, 50 μg/ml of kanamycin, 34 μg/ml of chloramphenicol, and 100 μg/ml of ampicillin.

### Construction of *Salmonella* mutant strains

The *Salmonella* mutant strains were constructed as previously described [23]. Briefly, prior to the target gene deletion events, the host strains were electroporated with the helper plasmid pKD46 (Addgene, USA), which provides the inducible Red lambda components required for recombination. The target genes were replaced with the chloramphenicol acetyltransferase cassette contained on a linear polymerase chain reaction (PCR) product amplified from the pKD3 (Addgene, USA) plasmid. Recombinant clones were selected by plating on LB agar containing 34 μg/ml of chloramphenicol (Sigma-Aldrich, USA). Successful gene deletions were confirmed using flanking and internal PCR primers shown in Table S2.

### Experimental co-adaptation of *Salmonella* with *T. thermophila*

The experiment was initiated from a single ancestral colony of *S.* Enteritidis SDC-11, which was isolated from diseased ducks in the Jiangsu province of China. The ancestral strain was cultured alone or co-cultured with *T. thermophila* SB210, with a predator-to-prey ratio of 1:3000, in 2 L conical flasks containing 1.5 L of Tris-buffered saline solution (TBSS) (2 mM KCl, 1 mM CaCl_2_, 0.5 mM MgCl_2_, and 1 mM Tris, pH 7.0). The conical flasks were kept at 28°C without shaking. Every 16 h, the co-culture was filtered through 8 μm filters (Millipore, USA), followed by submersing the filters in 5 ml TBSS to allow the EFVs of *T. thermophila* to release from the filter. Then, the filters were removed from the TBSS and drained. To eliminate any extracellular bacteria, the suspensions containing the EFVs were incubated with 300 μg/ml gentamicin at 28°C for 1 h, and the EFVs were collected by centrifugation at 3220 *g* for 20 min. After being washed three times in TBSS, the EFVs were incubated in LB broth at 37°C for 6 h to allow the growth and adaptation of *S.* Enteritidis [19]. At the end of each 16 h cycle, 90% of the EFVs were transferred into a new vial containing fresh TBSS to initiate the next round of co-adaptation. This iterative transfer process was continued to facilitate long-term adaptation under predation pressure. A total of five passages of cultures were performed. Each isolate was designated as "EFV" followed by a numeric suffix corresponding to the number of transmission generations it underwent. For example, "EFV1" refers to the *Salmonella* isolate from the first generation of EFVs, while "EFV2" refers to the isolate from the second generation. To ensure biological replication, three independent co-adaptation experiments were conducted. In the subsequent phenotype tests, a total of nine clones from each passage of EFVs were examined. These nine clones were derived from the three independent co-adaptation experiments, with three clones randomly selected from each experiment.

### Characterization of colony morphology

For colony morphology characterization, the EFVs collected from each generation were diluted 100-fold in sterile TBSS and then spread evenly onto LB agar plates supplemented with 1% (w/v) Congo red (CR) (Sigma-Aldrich, USA). Colonies were grown at 37°C for 48 h. Wild-type *S.* Enteritidis SDC-11 was used as a control strain, displaying characteristic white colonies on the CR plates.

### Motility assay

Swimming motility assays were performed on LB plates containing 0.4% (w/v) agar (Sigma-Aldrich, USA). A fresh single colony of the *Salmonella* isolates was inoculated into the center of the agar plates by a sterile stab. The plates were incubated at 37°C for 12 h. After incubation, the plates were imaged using a Gel Doc imaging system (Bio-Rad, USA), and the swimming diameter (mm) was measured to quantify the motility. The experiment was repeated independently more than three times to confirm the results.

### Auto-aggregation assay

Bacteria were grown in LB medium at 37°C in a shaking incubator until reaching the logarithmic phase and washed three times with phosphate-buffered saline (PBS). The bacterial suspension was then adjusted to an OD_600_ of 2.0 in a new tube with a final volume of 1 ml. To evaluate auto-aggregation, the cultures were left to stand at room temperature without agitation. After 6 h, 200 μl of the upper culture supernatant was carefully taken, and the OD_600_ value was measured using a spectrophotometer (Thermo Fisher Scientific, USA). The auto-aggregation percentage was calculated using the formula: auto-aggregation (%) = [(OD_600_ initial - OD_600_ supernatant) / OD_600_ initial] × 100. The experiment was repeated independently more than three times to confirm the results.

### Quantification of biofilm biomass

Biofilms were formed in microtiter plates (Corning, USA), and biomass was quantified by the crystal violet (CV) staining assay, as described previously [24]. Briefly, *Salmonella* clones were grown in LB to an OD_600_ of 1.0, which correlates to a bacterial concentration of 5 × 10^8^ colony-forming units (CFUs)/ml. The normalized suspensions were diluted 100-fold and inoculated into fresh LB and grown for 24 h at 37°C to allow biofilm formation. Following incubation, the aqueous phase was carefully removed, and the wells were washed three times with PBS. The CV solution (0.1%) was added to each well and incubated for 10 min at room temperature to stain the biofilm biomass. Excess CV was removed by washing the wells with sterile water. The bound CV, representing biofilm biomass, was solubilized using 95% ethanol. The OD_595_ value was measured using a microplate reader (Tecan Spark, Switzerland). Baseline values obtained from PBS were subtracted from all measurements to correct for background. The experiment was repeated independently more than three times to confirm the results.

### Cellulose production

Cellulose production in *S.* Enteritidis strains was determined quantitatively using the CR dye binding test. Colonies were suspended in PBS and adjusted to an OD_600_ of 1.0. A 1 ml aliquot of the suspension was transferred into a new tube containing 0.05% (w/v) CR. The mixture was incubated at 37°C for 2 h to allow the binding of CR to cellulose. Following incubation, the cells were removed by centrifugation at 6500 *g* for 10 min, and the supernatant was collected for further analysis. The unbound CR remaining in the supernatant was quantified by measuring the absorbance at 490 nm using a microplate reader. The amount of CR retained in the supernatant inversely correlates with the cellulose content in the bacterial strains. The experiment was repeated independently more than three times to confirm the results.

### Acid resistance

To evaluate acid resistance, overnight cultures of *S.* Enteritidis strains were grown in LB broth at 37°C with shaking at 180 rpm. The cultures were harvested by centrifugation and washed twice with sterile PBS. The bacterial suspensions were adjusted to an OD_600_ of 1.0 in PBS. Aliquots (1 ml) were transferred to PBS acidified to pH 5.5 with HCl and incubated at 37°C for 1 h without shaking. After incubation, 100 μl of the suspensions was serially diluted in PBS and plated on LB agar to determine viable CFU. The survival rate was calculated as the percentage of CFU compared to the initial count before acid exposure. The experiment was repeated independently more than three times to confirm the results.

### Oxidative stress resistance

To assess oxidative stress resistance, overnight cultures of *S.* Enteritidis strains were grown in LB broth at 37°C with shaking at 180 rpm. Bacterial cells were harvested by centrifugation and washed twice with sterile PBS. The bacterial suspensions were adjusted to an OD_600_ of 1.0 in PBS. Aliquots (1 ml) were incubated with H_2_O_2_ (a final concentration of 10 mM) at 37°C for 1 h without shaking. Following the incubation, 100 μl of the suspensions was serially diluted in PBS and plated on LB agar to determine viable CFU. The survival rate was calculated as the percentage of CFU compared to the initial count before oxidative stress exposure. The experiment was repeated independently more than three times to confirm the results.

### Transmission electron microscopy

To visualize bacterial morphology, *S.* Enteritidis strains were grown in LB broth for 16 h at 37°C. After incubation, bacterial suspensions were prepared and adsorbed on formvar–carbon-coated copper grids (300 mesh) for 5 min to allow adhesion. Following this, the grids were washed three times by transferring them onto droplets of distilled water to remove unbound particles. The samples were fixed using glutaraldehyde (prepared in 0.1 M sodium cacodylate buffer) to preserve cellular structure. Negative staining was performed by applying 1% sodium-phosphotungstic acid solution (pH 7.2) for 5 min to enhance contrast. Excess staining solution was carefully removed using filter paper. Prepared samples were examined under a Hitachi 600 transmission electron microscope (Hitachi, Japan), and images were captured to analyse bacterial ultrastructure.

### Scanning electron microscope

To observe bacterial surface structures, *S.* Enteritidis strains were grown to stationary phase in LB broth. Cultures were harvested by centrifugation at 4000 *g* for 5 min and washed three times with PBS to remove residual media. The bacterial suspensions were adjusted to an OD_600_ of 1.0. Cells were pelleted by centrifugation at 4000 *g* for 10 min and fixed by 2.5% glutaraldehyde for 3 h to preserve cellular morphology. Next, fixed samples were dehydrated with a water/ethanol gradient of 30%, 50%, 70%, 95%, and 100% ethanol, followed by treatment with absolute acetone for 30 min. After air-drying at room temperature, the specimens were coated with a thin layer of gold using a sputter coater and then examined using a Regulus 8100 scanning electron microscope (Hitachi, Japan) to capture high-resolution images of bacterial surface structures.

### Library construction for PacBio sequencing

The SMRTbell library was constructed using the SMRTbell Express Template Prep Kit 2.0 (Pacific Biosciences). Briefly, 5 μg of the genomic DNA was carried into the first enzymatic reaction to remove single-stranded overhangs, followed by treatment with repair enzymes to repair any damage that may be present on the DNA backbone. Then, the ends of the double-stranded fragments were polished and subsequently tailed with an A-overhang. Ligation with T-overhang SMRTbell adapters was performed at 20°C for 60 min. Following ligation, the SMRTbell library was purified with 1X AMPure PB beads. The size distribution and concentration of the library were assessed using the FEMTO Pulse automated pulsed-field capillary electrophoresis instrument (Agilent Technologies, Wilmington, DE) and the Qubit 3.0 Fluorometer (Life Technologies, Carlsbad, CA, USA). Following library characterization, 3 μg was subjected to a size selection step using the BluePippin system (Sage Science, Beverly, MA) to remove SMRTbells ≤25 kb. After size selection, the library was purified with 1X AMPure PB beads. Library size and quantity were assessed using the FEMTO Pulse and the Qubit dsDNA HS Assay kit. Sequencing primer and Sequel II DNA polymerase were annealed and bound, respectively, to the final SMRTbell library. The library was loaded at an on-plate concentration of 35 pM using diffusion loading. SMRT sequencing was performed using a single 8 M SMRT Cell on the Sequel II System with Sequel II Sequencing Kit, 900-min movies by Frasergen Bioinformatics (Wuhan, China).

### Genome assembly

The genome assembly of *S.* Enteritidis strains was conducted using continuous long reads generated from SMRT sequencing. All subread data were utilized to construct the draft genome assembly using mecat2 (v20190226) with the default parameters. To improve the accuracy of the draft assembly, a multi-step error correction process was employed. First, the gcpp in the SMRT link toolkit was performed to correct errors after the initial assembly of the genome. Second, two datasets of short reads (4.34 Gb and 3.77 Gb) were used to refine the assembly further with Pilon (v1.22) [25]. Due to the heterozygosity of the genome, Haplotigs purge was used to filter redundant sequences [26].

### Bacterial resequencing

To investigate genomic changes in *S.* Enteritidis strains derived from EFVs, whole-genome resequencing was performed. Genomic DNA was extracted from EFV isolates (EFV1 to EFV5). The quality and concentration of DNA were assessed using a NanoDrop spectrophotometer and a Qubit fluorometer to ensure high-purity samples suitable for sequencing. Sequencing libraries were prepared with the Nextera XT DNA Library Prep Kit (Illumina, USA). Genomic DNA was fragmented, adapters were ligated, and libraries were amplified according to the manufacturer’s guidelines. The library quality and fragment size distribution were verified using an Agilent 2100 Bioanalyzer. Paired-end sequencing (150 bp reads) was conducted on a NovaSeq 6000 System (Illumina), achieving a minimum of 100× coverage per sample to ensure sufficient depth for accurate variant analysis. Raw sequencing data were processed to remove adapters and low-quality reads using Trimmomatic (v0.39) [27], and the cleaned reads were validated using Fastp [28]. High-quality reads were aligned with the SDC-11 genome using BWA-MEM (v0.7.17) [29]. Variants—including single-nucleotide polymorphisms (SNPs) and small insertions/deletions (INDELs)—were identified with GATK (v4.2) [30] and annotated using SnpEff (v5.0) [31]. Structural variants—such as large deletions, duplications, or inversions—were detected and verified through manual curation.

### Lipopolysaccharide profiles

Lipopolysaccharide (LPS) was extracted using the LPS Extraction Kit (iNtRON, South Korea). A single colony from different bacterial strains was cultured in 4 ml of LB broth until the OD_600_ reached 0.8–1.2. Cells were harvested by centrifugation at 13000 *g* for 2 min, and the pellet was resuspended to ensure efficient lysis. A total of 1 ml of lysis buffer was added and mixed thoroughly, followed by the addition of 200 μl of chloroform to remove RNA and protein contaminants. After vigorous mixing, the suspension was incubated at room temperature for 5 min and centrifuged at 13000 *g* for 10 min at 4°C. The supernatant (400 μl) was transferred to a new tube, and 800 μl of purification buffer was added. The mixture was incubated at −20°C for 10 min and then centrifuged at 13000 *g* for 15 min at 4°C to obtain the LPS pellet. The pellet was washed with 70% ethanol, dried at room temperature, and dissolved in 50 μl of 10 mM Tris-HCl buffer (pH 8.0). To reduce protein contamination, the LPS sample was incubated with 1 μl of proteinase K (25 mg/ml stock) at 50°C for 30 min. The extracted LPS was mixed with sodium dodecyl sulfate (SDS) loading buffer, followed by boiling for 5 min. The LPS samples were analyzed by SDS-polyacrylamide gel electrophoresis (SDS-PAGE) on a 12% (w/v) acrylamide gel followed by silver staining.

### β-galactosidase activity

The β-galactosidase activity was determined as previously described [32], with a modification. Bacteria containing the lacZ reporter plasmid were inoculated in 1 ml of LB broth until the OD_600_ reached 1.0. Cultures were harvested by centrifugation, and the bacterial pellet was washed three times with Z buffer (60 mM Na_2_HPO_4_, 40 mM NaH_2_PO_4_, 10 mM KCl, and 1 mM MgSO_4_, pH 7.0) and resuspended to an OD_600_ of 1.0 with Z buffer. For each assay, 200 μl of bacterial suspension was transferred to a new tube, and 3.5 μl of β-mercaptoethanol, 6.5 μl of 0.1% SDS, and 1.25 μl of chloroform were added. The mixture was vortexed briefly and incubated at 30°C for 5 min to lyse the cells. Following this, 100 μl of O-nitrophenyl-β-D-galactopyranoside (4 mg/ml) was added as a substrate, and the reaction was allowed to proceed at 30°C until the solution turned yellow, indicating the completion of the reaction. The reaction was then terminated by adding 200 μl of termination solution (1 M Na_2_CO_3_), and the reaction time (RT, in minutes) was recorded. The suspension was then centrifuged at 12000 *g* for 10 min at 4°C. Then, 200 μl of the clarified supernatant was transferred to a new 96-well microtiter plate for measurement of the absorbances at 420 nm (OD_420_) and 550 nm (OD_550_) using a microplate reader. The β-galactosidase activity was calculated in Miller Units (MU) using the following formula: MU = 1000 × (OD_420_–1.75 × OD_550_) / (RT × V × OD_600_), where RT is the reaction time in minutes, V is the total sample volume in milliliters, and OD_600_ is the optical density of the culture at 600 nm. The experiment was repeated independently more than three times to confirm the results.

### Real-time quantitative PCR

Real-time quantitative RT-qPCR was performed to measure the transcription levels of selected genes using the primers listed in Table S2. Total RNA was extracted from stationary-phase bacteria using the E.Z.N.A. Bacterial RNA Isolation Kit (Omega, Beijing, China), following the manufacturer’s protocol. RNA quality and concentration were assessed using a NanoDrop spectrophotometer to ensure high-purity RNA suitable for downstream analysis. Reverse transcription of 1 μg total RNA was performed using HiScript II QRT Supermix (Vazyme Biotech, Nanjing, China) to synthesize cDNA. Quantitative PCR was carried out using the One Step RT-qPCR SYBR Green Kit (Vazyme Biotech) on an Applied Biosystems StepOnePlus Real-Time PCR System (96-well format). Each reaction contained 5 μl of diluted cDNA, 10 μl of SYBR Green Master Mix, 0.4 μl of each primer (10 μM), and nuclease-free water to a final volume of 20 μl. The housekeeping gene 16S rRNA was used as an internal control to normalize target gene expression levels. The fold change of mRNA transcription levels was calculated using the 2^−ΔΔ*C*t^ method. The experiment was repeated independently more than three times to confirm the results.

### Predation resistance to *T. thermophila*

The anti-predation ability of different *S.* Enteritidis strains was assessed by detecting the relative survival of the bacteria after co-culture with *T. thermophila* as previously described [33] with slight modifications. After overnight incubation at 28°C and 180 rpm, the bacterial cells were harvested by centrifugation at 6500 *g* for 5 min, and the pellets were washed three times with TBSS. Then, the bacterial concentration was adjusted to 1 × 10^9^ CFU/ml. *T. thermophila* SB210 with an initial inoculum of 1 × 10^3^ cells/ml was cultured in SPP medium at 28°C for 36 h, and then the cells were harvested by centrifugation at 2000 *g* for 10 min. After being washed three times with TBSS, the pellets were adjusted to 2 × 10^5^ cells/ml. The suspensions from the bacteria and *T. thermophila* were mixed at a volume ratio of 1:1 and then added into 96-well cell plates at 100 μl per well. The suspensions containing *Salmonella* strains mixed with the equal volume of TBSS were used as a control. Furthermore, TBSS served as a blank control alone. The 96-well plates were incubated at 28°C for 12 h, and the OD_450_ was measured. The relative survival of the bacteria was expressed as the OD_450_ value of the bacterial suspensions co-cultured with *T. thermophila* divided by the OD_450_ value of the bacterial suspensions alone. The experiment was repeated independently more than three times to confirm the results.

### RNA-Seq and data analysis

RNA-Seq (quantification) was used to study gene expression and to provide accurate digital expression profiles. The clean reads were mapped to the reference genome using HISAT2 (v2.0.4) (http://www.ccb.jhu.edu/software/hisat/index. shtml) [34]. The clean reads were aligned with the reference gene set by Bowtie2 (v2.2.5) (http://bowtiebio.sourceforge.net/%20Bowtie2%20/ index.shtml) [35], and the gene expression level was calculated using featureCounts (subread) v2.0.1 [36]. The genome of the wild-type *S.* Enteritidis SDC-11 was used as the reference genome to construct the necessary database for enrichment.

Differential gene expression analysis was performed using the package DESeq2 [37] in the R environment. Raw read counts obtained from RNA-Seq data were used as input for DESeq2, which normalizes the data and identifies the differentially expressed genes (DEGs) based on statistical modeling. Genes with an adjusted *P-*value (false discovery rate) of <0.05 and a fold change threshold of >2 or < 0.5) were considered significantly differentially expressed. The results included log2 fold change values, *P-*values, and adjusted *P-*values for each gene, providing robust statistical control for multiple testing. Identified DEGs were subsequently subjected to downstream analyses (Table S3)—including functional annotation, pathway enrichment, and co-expression network construction—to explore their biological significance.

Kyoto Encyclopedia of Genes and Genomes (KEGG) (https://www.kegg.jp/) [38] was used to determine the enrichment of expressed genes using Phyper. The significance levels of the KEGG terms and pathways were corrected using a *Q*-value with a strict threshold (*Q*-value ≤0.05). Using Weighted Gene Co-expression Network Analysis (WGCNA) [39], some potential key genes associated with the adaptive evolution of *Salmonella* within *T. thermophila* were explored by constructing an interrelated gene network and grouping highly co-expressed genes into modules based on the transcriptome data of the wild-type (WT) and EFV5 strains.

### Electrophoretic mobility shift assay

Electrophoretic mobility shift assay (EMSA) was performed as previously described [40]. The primers used to amplify the *csgD* gene are listed in Table S2. Target DNA fragments were amplified using primer pairs designed for specific regulatory regions (300 to 500 bp), and a 200 bp DNA fragment was used as the negative control. The purified protein (0.2–1.0 μM) was incubated with the DNA fragment (25 nM) in binding buffer [20 mM Tris-HCl (pH 7.5), 30 mM KCl, 1 mM DTT, 1 mM EDTA (pH 7.5), and 10% (v/v) glycerol] in a final volume of 20 μl for 30 min at 37°C. Following incubation, the samples were loaded on a 10% polyacrylamide gel and electrophoresed in 0.5 × TBE (44.5 mM Tris, 44.5 mM boric acid, and 1 mM EDTA, pH 8.0) under 120 V for 1.5 h in an ice bath to prevent overheating. The gel was stained using Gold nucleic acid staining solution for 10 min to visualize DNA–protein complexes. The stained gel was observed and documented under UV transillumination using the Gel Doc XR (Bio-Rad, CA, USA).

### Quantification of cyclic diguanylate monophosphate (c-di-GMP)

Measurement of intracellular c-di-GMP level was performed using a c-di-GMP ELISA Kit (Cayman Chemical, USA). Briefly, bacterial cells were cultured to the logarithmic growth phase under optimal conditions. After being harvested by centrifugation at 3000 *g* for 10 min, the cell pellet was washed with cold PBS to remove any residual media. The washed cells were then resuspended in a lysis buffer (20 mM Tris-HCl (pH 7.5) containing 1 mM phenylmethanesulfonyl fluoride) and subjected to mechanical disruption via sonication to ensure complete cell lysis. The resulting lysate was centrifuged at 12000 *g* for 10 min at 4°C to remove cell debris, and the clarified supernatant was collected for further processing according to the manufacturer's instructions. The absorbance was measured at 450 nm to determine the c-di-GMP concentration based on the established standard curve. The experiment was repeated independently more than three times to confirm the results.

### Statistical analysis

Statistical analyses were conducted using GraphPad Prism version 9 (www.graphpad.com). For experiments with multiple samples, including motility, auto-aggregation, biofilm biomass, cellulose production, acid resistance, oxidative stress resistance, predation resistance, and gene transcription, one-way analysis of variance (ANOVA) was used for the analysis. For the β-galactosidase activity, two-tailed Student’s *t*-tests were used to compare means between experimental samples and controls. For comparisons among multiple groups, two-way ANOVA followed by Sidak’s multiple comparisons test was performed. Specific details regarding the statistical tests applied to each dataset are provided in the figure captions. All bioinformatic statistical analyses, e.g. DEG identification, KEGG enrichment, and WGCNA, were conducted using the R software (version 4.2.2). Visualization of KEGG pathway enrichment results was performed using Chiplot (https://www.chiplot.online), ensuring clear and interpretable graphical representations.

## Results

### Expelled food vacuole-adapted *Salmonella* strains enhance environmental adaptability

**Figure 1:**
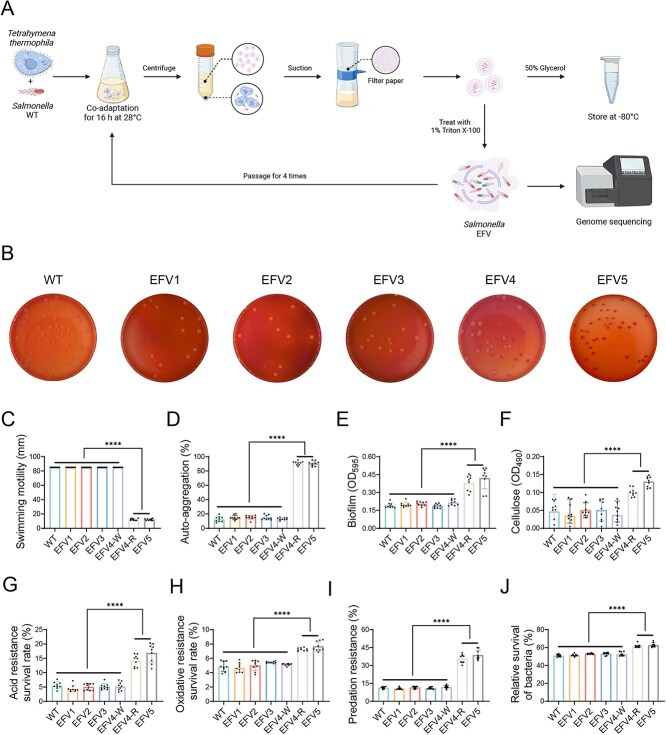
Enhanced environmental adaptability of EFV-adapted strains. (A) Schematic representation of the experimental protocol used for the co-adaptation of *S.* Enteritidis SDC-11 with *T. thermophila* SB210. Bacteria were co-incubated with *T. thermophila* for 16 h at 28°C, followed by centrifugation and filtration to collect the expelled food vesicles (EFVs). This process was continued for a total of five passages, and bacteria from EFVs were isolated on LB agar and designated EFV1 to EFV5. The co-adaptation experiment was repeated in triplicate. (B) Colony morphology of *Salmonella* strains. Colonies were grown on CR plates at 37°C for 48 h. The strains, from EFV1 to EFV3, showed no changes in colony morphology, similar to the wild-type strain with white colonies. EFV4 had two distinct types of colonies: white (W) and red (R). EFV5 only exhibited red colonies. (C) Swimming motility of *Salmonella* strains. After 12 h of culture, bacterial motility was assayed on LB soft agar plates containing 0.4% (w/v) agar. (D) Auto-aggregation ability of *Salmonella* strains. Auto-aggregation was evaluated by measuring the OD_600_ value of the supernatant after static incubation of bacterial cultures (OD_600_ = 2.0) at room temperature for 6 h. (E) Biofilm formation ability of *Salmonella* strains. Biofilm formation was quantified using crystal violet (CV) staining in microtiter plates. Biofilms were solubilized with ethanol, and the OD_595_ value was recorded. (F) Cellulose production of *Salmonella* strains using Congo red (CR) binding. The mixture with bacterial suspensions and 0.05% (w/v) CR was incubated at 37°C for 2 h, and the OD_490_ value of the supernatant was measured. (G) Acid resistance of *Salmonella* strains. The bacterial survival in acidified PBS (pH 5.5) for 1 h was determined. (H) Oxidative stress resistance of *Salmonella* strains. The bacterial survival was determined after incubated with 10 mM H_2_O_2_ for 1 h. (I) Predation resistance of *Salmonella* strains against *T. thermophila*. (J) Relative survival of *Salmonella* strains after exposure to *T. thermophila* predation. For each phenotype, a total of nine clones from three independent co-adaptation experiments were assayed. Error bars represent the standard deviations (*n* = 9) for all the plots. One-way ANOVA test was used to determine the statistical significance between the groups. ^****^P < 0.0001.

To assess the impact of predation pressure imposed by *T. thermophila* on the adaptive traits of *Salmonella*, a continuous serial passaging was conducted, as illustrated in Fig. 1A. Bacteria were collected from *T. thermophila* EFVs 16 h after co-incubation and then exposed again to *T. thermophila* for further passaging. This time point was selected on the basis of a preliminary observation, which showed significantly increased EFV production after co-incubated with *S.* Enteritidis SDC-11. Our data revealed that the former populations (EFV1–EFV3) after passage through *Tetrahymena* exhibited identical white colonies on the CR plates, but in EFV4 populations, two different types of cells were observed: one forms a white colony (W strain), and the other forms a red colony (R strain). In all EFV5 colonies, cells exhibit the red colony morphology (Fig. 1B). Correspondingly, EFV1–EFV3 have not exhibited any alterations in the other phenotypes tested, whereas the EFV4-R strain and all the EFV5 showed consistent phenotypic changes, manifested as a substantial reduction in motility (Fig. 1C), and significantly enhanced auto-aggregation (Fig. 1D), biofilm formation (Fig. 1E), and cellulose production (Fig. 1F). Furthermore, both acid resistance (Fig. 1G) and oxidative stress resistance (Fig. 1H) were enhanced in EFV4-R and EFV5 strains, suggesting an improved ability of the strains to survive hostile environmental conditions. Similarly, when exposed to *Tetrahymena*, predation resistance (Fig. 1I) and survival ability (Fig. 1J) of EFV4-R and EFV5 in food vacuoles were markedly increased. Images obtained from transmission electron microscopy (Fig. S1A) indicated that, different from the WT and former populations, EFV4-R and EFV5 showed slightly uneven surface and significantly damaged cell membranes, as observed at one end of the bacterial cells. These structural changes may lead to increased sensitivity of the bacterial membrane to environmental stress, as previously described [41]. For the scanning electron microscopy images (Fig. S1B), WT showed a regular rod-like shape with a smooth surface, while the EFV5 displayed uneven undulations on the surface, although maintaining a rod-shaped morphology. Collectively, these results indicate that prolonged exposure to *T. thermophila* leads to adaptive phenotypic changes in *Salmonella*, particularly in the EFV4-R and EFV5 strains, potentially contributing to increased environmental persistence.

### Gene *rfbP* plays a crucial role in the adaptive evolution of *Salmonella* upon *Tetrahymena* predation pressure

**Figure 2:**
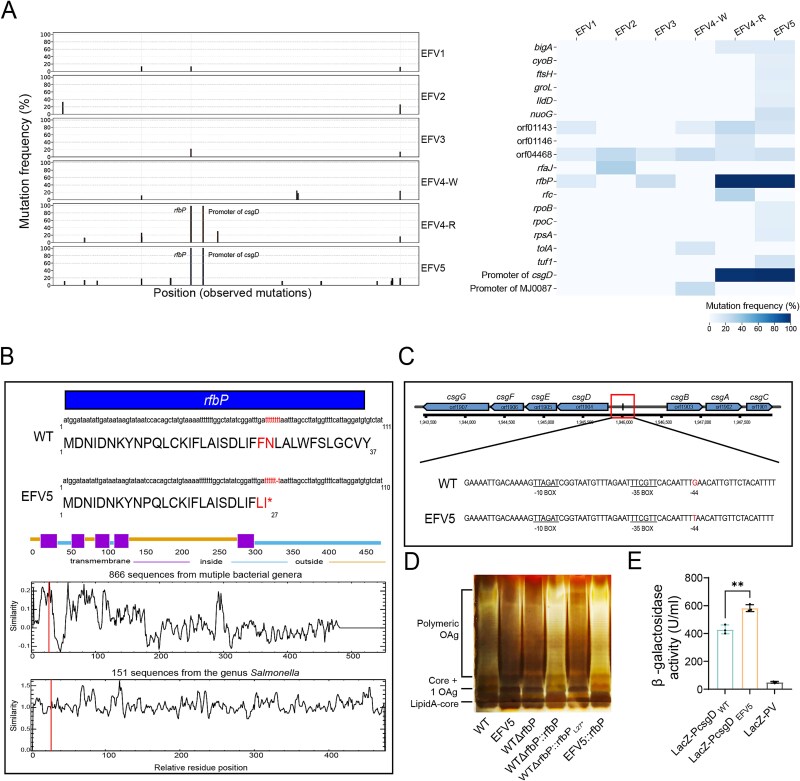
Whole-genome sequencing reveals key mutations in the EFV strains. (A) Mutation frequencies in the EFV strains compared to WT. Mutations were identified through whole-genome sequencing and mapped to the coding and intergenic regions. Frequencies of SNPs and INDELs are shown across the genome (left panel), with a heatmap summarizing affected genes (right panel). (B) Analysis of the *rfbP* sequence. EFV5 contains a one-base deletion in the *rfbP* gene, which introduces a stop codon at position 27. A total of 866 RfbP amino acid sequences are selected for similarity analysis, among which 151 are from the genus *Salmonella*, revealing significant intergenus variability but high conservation within the genus *Salmonella*. Amino acid position 27 is indicated by a vertical line. (C) Promoter region analysis of *csgD* in the EFV5. The genomic region encompassing upstream regulatory elements of *csgD* was compared between the WT and EFV5 strains. A single mutation was identified at position −44 within the *csgD* promoter region in EFV5. (D) LPS profiles of WT, EFV5, and mutants visualized using silver-stained SDS-PAGE. LPS samples from equal numbers of bacterial cells were loaded in each lane and analyzed by SDS-PAGE on a 12% (w/v) acrylamide gel followed by silver staining. (E) β-galactosidase activity of *csgD* promoter constructs. Reporter plasmids containing the *csgD* promoter sequences of wild-type and EFV5 strains were transformed into the WT strain, respectively, and enzyme activities were quantified. The empty plasmid (LacZ-PV) serves as a negative control. The *t*-test was used to determine the statistical significance in β-galactosidase activity between the groups. ^*^P < 0.05, ^**^P < 0.01.

To elucidate the genetic changes associated with the evolution of EFVs under *T. thermophila* predation pressure, we conducted a detailed whole-genome sequencing analysis for EFV populations of different passages. The sequencing depth ranged from 120.5× to 182.25×, with an average of 147.46×. Genes with mutation rates greater than 10% for all generations are shown in Table S4. As shown in Fig. 2A, some mutations—including SNPs and INDELs—were identified within different passages of EFVs compared with the WT strain, most of which occur in genes involved in LPS synthesis (such as *rfaJ* and *rfbP*) and prophage (such as orf01143, orf01146, and orf04468). For the prophage genes, the majority of observed mutations are synonymous and do not affect the integrity of the encoded proteins. In each generational passage, certain unique genetic mutations may confer additional adaptive advantages to the bacteria in response to environmental pressures. For instance, in EFV2, a mutation at position 125 921 of the *rfaJ* gene reached a frequency of 32.57%, potentially altering the core structure of LPS [42]. In EFV4-R, the mutation at position 2 135 793 of the *bigA* gene reached a frequency of 30%, suggesting a possible role in bacterial colonization [43]. By EFV5, mutations in *tuf1*, *nuoG*, and genes encoding sigma factors highlight distinctive adaptations of this generation to predatory pressure from protozoa [44–46]. These high-frequency mutation genes play a key role in the adaptation of EFV5 to predation pressure, suggesting that bacteria optimize their biological response through mutations in these genes. The evolutionary trajectory of the *rfbP* gene reveals substantial shifts in mutation frequency across generations. In EFV1, a nonsynonymous SNP was detected at position 1 789 434 of the *rfbP* gene, with a mutation frequency of 12.94%. By EFV3, a subsequent mutation at position 1 788 380 in the *rfbP* gene increased to a frequency of 21.64%, reflecting its growing significance in the adaptation process. In EFV4-R and EFV5, the mutation at position 1 788 404 of the *rfbP* gene reached fixation (100%), indicating that this mutation has become fully established in these variants. This progressive rise in mutation frequency highlights the central role of *rfbP* in bacterial evolution and environmental adaptation. The EFV5 generation exhibited the highest number of multi-locus mutations, involving 14 genes, with a mutation within the *csgD* promoter region being particularly noteworthy. This mutation in the promoter region of *csgD* reached a frequency of 98.3% in EFV4-R, which subsequently increased to 100% in EFV5. This fixation of point mutation indicates a significant adaptive advantage for EFV5. The synergistic effects of these multi-locus mutations may collectively enhance the survival and competitive capacity of EFV5 under environmental pressures.

We focused on exploring two high-frequency mutation sites in EFV5. One occurred at the 74th nucleotide position (1788404) of the *rfbP* gene (orf01730), encoding undecaprenyl-phosphate galactose phosphotransferase, a key enzyme in glucose metabolism and LPS synthesis [47]. This mutation resulted in the substitution of the 27th amino acid from alanine to a stop codon, truncating protein translation (Fig. 2B). We selected 866 RfbP amino acid sequences from various bacteria for similarity analysis, including *Pseudomonas aeruginosa*, *E. coli*, *Klebsiella pneumoniae*, *Vibrio parahaemolyticus*, and multiple *Salmonella* serotypes, revealing significant intergenus variability but high conservation within the genus *Salmonella* (Fig. 2B). Another mutation was found in the promoter region (1946008) of the *csgD* gene (Fig. 2C), a transcriptional factor that plays a major role in regulating biofilm formation and bacterial community behavior in *Salmonella* [48]. The G-to-T base transversion at position 44 within the *csgD* promoter region has been reported to influence the transcriptional level of *csgD*, thereby enhancing bacterial biofilm formation in *Salmonella. typhimurium* [49]. To investigate the relationship between the two mutations and altered phenotypes in the EFV5, we constructed the *rfbP* (WTΔ*rfbP*) and the *csgD* (WTΔ*csgD*) deletion mutants in the WT background and used them as a basis to build two point mutation strains, WTΔ*rfbP*::*rfbP*_L27*_ and WTΔ*csgD*::*csgD*_-44G > T_. Before examining the phenotypic traits, we analyzed the LPS profile by silver-stained SDS-PAGE because of the important role of *rfbP* in O-antigen synthesis. LPS purified from the WTΔ*rfbP*, WTΔ*rfbP*::*rfbP*_L27*_, and EFV5 displays similar profiles (Fig. 2D), revealing the distribution of bands in the lower region of the gel with a lack of the bands located in the upper region of the gel, which is indicative of a high molecular weight O-antigen. Meanwhile, overexpression of the intact *rfbP* gene in EFV5 restored the O-antigen bands to the WT level. These observations indicate that this mutation of *rfbP* severely impairs LPS O-antigen synthesis in the EFV5. In addition, we measured the transcriptional activity of the original and mutated *csgD* promoters using a β-galactosidase assay. Our data showed that the mutated promoter region of *csgD* in the WT exhibited 36.7% higher activity than the original sequence (Fig. 2E), indicating enhanced *csgD* promoter activity due to the mutation.

### Deficiency of the *rfbP* greatly contributes to the adaptive evolution of *Salmonella* upon *Tetrahymena* predation pressure

**Figure 3:**
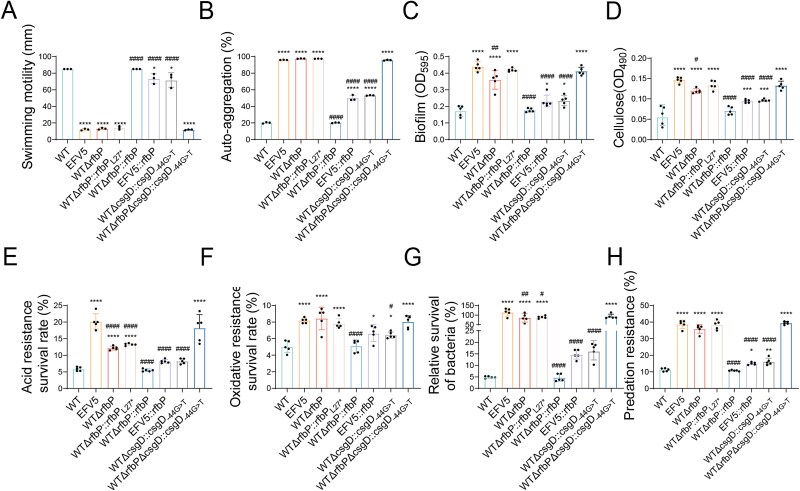
Phenotypic characterization of WT, EFV5, and mutant strains. (A) Bacterial swimming motility on LB soft agar plates. (B) Bacterial auto-aggregation ability. (C) Bacterial biofilm formation ability. (D) Cellulose production in *Salmonella* strains. (E) Bacterial acid resistance. (F) Oxidative stress resistance of *Salmonella* strains. (G) Predation resistance of *Salmonella* strains against *T. thermophila*. (H) Relative survival of *Salmonella* strains after exposure to *T. thermophila* predation. Error bars represent the standard deviations (*n* = 5) for all the plots. Statistical significance was determined using a one-way ANOVA test. *P < 0.05, **P < 0.01, ^***^P < 0.001, or ^****^P < 0.0001 indicates a significant difference between the designated strain and the WT strain. #*P <* 0.05, ##*P <* 0.01, or ####*P <* 0.0001 indicates a significant difference between the designated strain and the EFV5 strain.

To evaluate the contribution of two hotspot mutations to altered phenotypes of EFV5, we examined the phenotypic traits of different strains. We found that WTΔ*rfbP* and WTΔ*rfbP*::*rfbP*_L27*_ exhibited nearly identical phenotypic traits (Fig. 3). This is not difficult to understand because the site mutation of *rfbP* might lead to the termination of protein translation, which is equivalent to gene deletion. This speculation is further supported by similar LPS profiles from WTΔ*rfbP* and WTΔ*rfbP*::*rfbP*_L27*_ by silver-stained SDS-PAGE. Phenotypic changes caused by single mutation or deletion of *rfbP* and double mutations of *rfbP* and *csgD* (WTΔ*rfbP*Δ*csgD*::*csgD*_-44G > T_) in the WT are similar to those in the EFV5, while complementation of the *rfbP* gene in the WTΔ*rfbP* strain fully restored the aforementioned phenotypes to the WT levels. Furthermore, the phenotypic levels of the WTΔ*csgD*::*csgD*_-44G > T_ strain were comparable to those of the *rfbP*-complemented EFV5 strain (EFV5:: *rfbP*), and this phenotypic change was statistically significant compared to the WT, but the magnitude of the change was far less than that of EFV5. This finding indicates that a mutation confined solely to the *csgD* promoter region was insufficient to induce the phenotypic levels similar to those observed in EFV5. All the data provide evidence that mutation of *rfbP* is mainly responsible for the alteration in phenotypic traits of the EFV5.

### Mutation of *rfbP* is the main contributing factor to global transcriptomic changes of the EFV5

**Figure 4:**
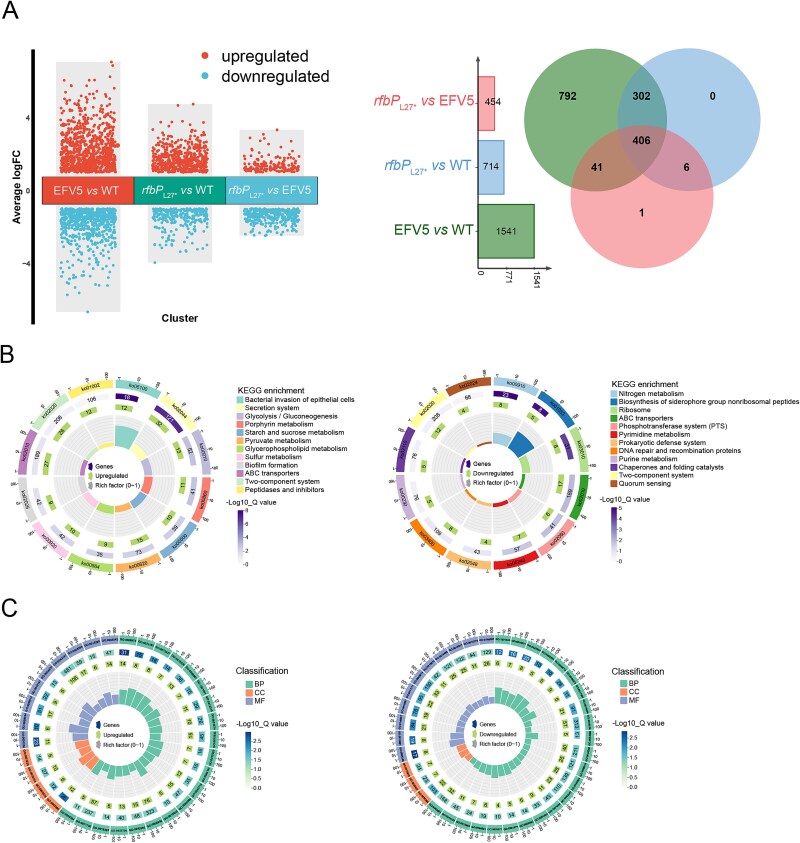
Transcriptomic analysis and functional enrichment of differentially expressed genes (DEGs) in WT, EFV5, and WTΔ*rfbP*::*rfbP*_L27*_. (A) Differential expression analysis showing the number of upregulated and downregulated genes across three comparisons: EFV5 *vs* WT, WTΔ*rfbP*::*rfbP*_L27*_  *vs* WT, and WTΔ*rfbP*::*rfbP*_L27*_  *vs* EFV5. The Venn diagram illustrates the overlap of DEGs among the comparisons, while the bar plot depicts the number of DEGs. (B) KEGG pathway enrichment analysis for the DEGs identified in each comparison. Circular bar plots display enriched pathways, including bacterial metabolism, secretion systems, and biofilm formation, with color gradients representing the -log10 *Q* values for significance and bar height indicating the number of genes involved in each pathway. (C) Gene Ontology (GO) classification for the DEGs from biological process (BP), cellular component (CC), and molecular function (MF) categories. Circular bar plots represent the number of genes, the fold enrichment, and the statistical significance (-log10 *Q* values) for each GO term.

To dissect the significance of the *rfbP* mutation in the EFV5, we performed a transcriptome sequencing of the WT, EFV5, and WTΔ*rfbP*::*rfbP*_L27*_ strains. Our data revealed that EFV5 had the highest number of the DEGs, with 1541 (858 upregulated and 683 downregulated) compared to the WT, accounting for 33.18% of the genome (Fig. 4A). The other two comparisons were performed between WTΔ*rfbP*::*rfbP*_L27*_ and WT, and between WTΔ*rfbP*::*rfbP*_L27*_ and EFV5, and identified 714 and 454 DEGs, respectively. There was an overlap of 708 DEGs between the two sets of DEGs (EFV5 *vs* WT and WTΔ*rfbP*::*rfbP*_L27*_  *vs* WT), and for the DEGs identified in the WTΔ*rfbP*::*rfbP*_L27*_, only 6 DEGs were not shared by the EFV5 (Fig. 4A).

KEGG pathway enrichment analysis showed that most of the DEGs shared by EFV5 and WTΔ*rfbP*::*rfbP*_L27*_ are linked to bacterial metabolism (Fig. 4B). Significantly upregulated pathways are related to bacterial metabolic pathways and secretion systems, indicating that the EFV5 strain might enhance its adaptability to environmental stress through metabolic reprogramming. Pathways related to biofilm formation and two-component systems (TCSs) were significantly enriched, consistent with the observed enhancement in biofilm formation, suggesting that the EFV5 strain possesses enhanced structural and functional adaptability under external pressure. In contrast, the downregulated genes were mainly involved in nitrogen metabolism, cofactor biosynthesis for non-ribosomal peptide synthesis, and the phosphotransferase system. Additionally, pathways related to ribosome synthesis (ribosome, pyrimidine metabolism, and purine metabolism) and DNA repair (DNA repair and recombination proteins) were enriched in the downregulated set, indicating that the EFV5 strain may adjust its genetic stability and nutrient acquisition mechanisms in response to environmental stress. These findings emphasize that bacteria can optimize their survival strategies by regulating metabolic pathways to cope with external environmental changes.

Gene Ontology (GO) enrichment analysis showed that most of the DEGs in the EFV5 strain were enriched in the biological process (BP) category (Fig. 4C), indicating that adaptive changes are mainly achieved by regulating BPs. Compared to the WT strain, genes upregulated in EFV5 were primarily involved in BPs such as stress response, metabolism, and transport. The enrichment of genes related to osmotic stress response (GO:0006970) suggests that EFV5 has the ability to adapt to or tolerate osmotic stress. In terms of metabolism, genes involved in iron–sulfur cluster assembly (GO:0016226) and ketone body synthesis (GO:0042181) reflect shifts in metabolic regulation and energy acquisition that may help EFV5 survive under nutrient-limited or stressful conditions. Regarding material transport, genes involved in amino acid transport (GO:0015833) and organic substance transport (GO:0071702) were also enriched, suggesting that EFV5 may have enhanced its ability to absorb and utilize available nutrients from the environment. Furthermore, the enrichment of genes related to cell structure—such as those involved in cell projection formation (GO:0042995) and biofilm formation (GO:0042710)—showed that EFV5 may enhance its ability to colonize the host or to adapt to a new environment through these processes.

The downregulated genes in EFV5 were largely enriched in BPs such as protein metabolism, energy metabolism, and nucleic acid metabolism. Among them, genes associated with protein metabolism (GO:0019538) were identified, indicating that EFV5 may optimize protein synthesis and degradation to adapt to environmental changes. In terms of energy metabolism, genes related to adenosine triphosphate (ATP)-dependent activity (GO:0140657) and ATPase activity (GO:0016887) were downregulated, reflecting adjustments in the energy consumption strategy in the EFV5. Genes involved in mRNA metabolism (GO:0016071) and DNA-templated transcription termination (GO:0006353) were downregulated, suggesting that the EFV5 regulates these processes to better adapt to environmental stress.

These findings reveal potential molecular strategies employed by the EFV-adapted strain to acclimate to the host environment, including the enhancement of stress response mechanisms, the fine-tuning of metabolic pathways, the optimization of nutrient absorption, and the adjustment of cellular structure and function. Moreover, the mutation of *rfbP* is the main contributing factor to the transcriptional changes of EFV5.

### Gene *csgD* is predicted to play a central regulatory role in enhancing the adaptation of *Salmonella* to predation pressure

**Figure 5:**
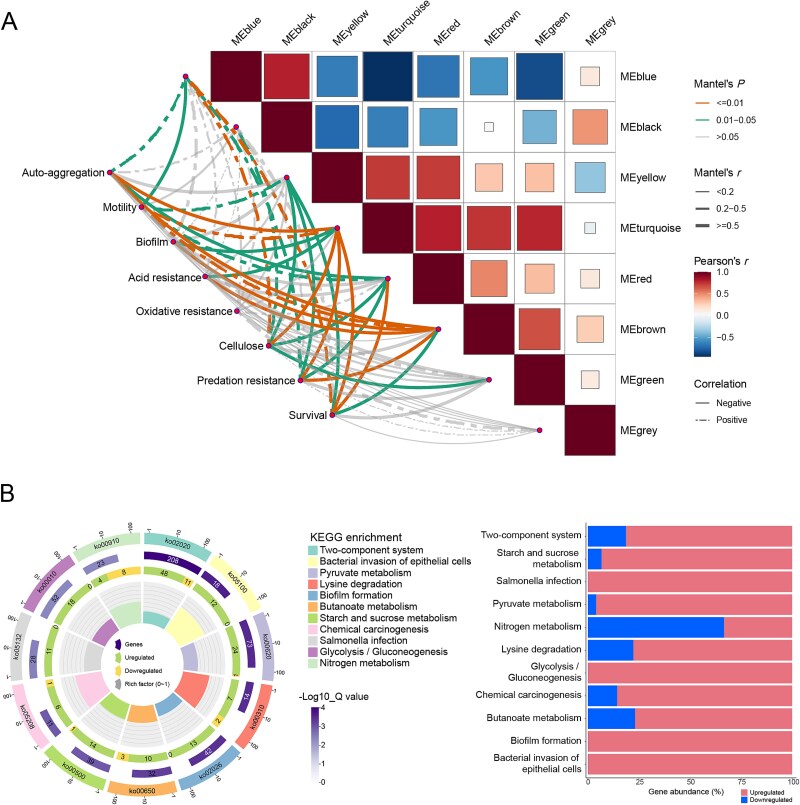
Weighted gene co-expression network analysis (WGCNA) and pathway enrichment analysis of identified gene modules in WT and EFV5 strains. (A) WGCNA analysis of gene expression in *Salmonella* after adaptation to *T. thermophila*. Genes were grouped into eight color-coded modules (MEblue, MEblack, MEyellow, MEturquoise, MEred, MEbrown, MEgreen, MEgrey) based on co-expression patterns. Correlation matrices were generated to evaluate the associations between gene modules and phenotypic traits, with Mantel’s *P* values and Pearson’s correlation coefficients determined. The phenotypic trait network was constructed to represent the associations between gene modules and phenotypic responses, with line types and thicknesses indicating correlation strength and direction. (B) KEGG pathway enrichment analysis for the MEblue module. The circular bar plot on the left illustrates the enriched pathways, displaying the number of upregulated and downregulated genes, along with the rich factor for each pathway. The bar plot shows the proportion of upregulated and downregulated genes across pathways, including those related to metabolism, biofilm formation, and cellular processes. The list of genes in the MEblue module is shown in Table S5.

By WGCNA, we explored the potential key genes that may reveal the mechanism underlying the adaptive evolution of *Salmonella* within *T. thermophila*. We identified eight color-coded modules and found that all phenotypic traits except for mobility exhibit a positive correlation between the MEblue and MEblack modules (Fig. 5A). The MEblue module demonstrates a more pronounced correlation with the phenotypic traits.

KEGG pathway analysis of the MEblue module revealed significant enrichment in biological pathways, such as the TCS, bacterial invasion of epithelial cells, pyruvate metabolism, lysine degradation, and biofilm formation (Fig. 5B). As for the TCS, approximately 69.6% of the genes are upregulated, indicating its significant role in response to predation pressure. Similarly, genes associated with biofilm formation also show a strong upregulation pattern, consistent with the phenotypic observation. On the contrary, 30.4% of the genes from the nitrogen metabolism pathway are downregulated, suggesting that nitrogen metabolism might be suppressed in the EFV5.

**Figure 6:**
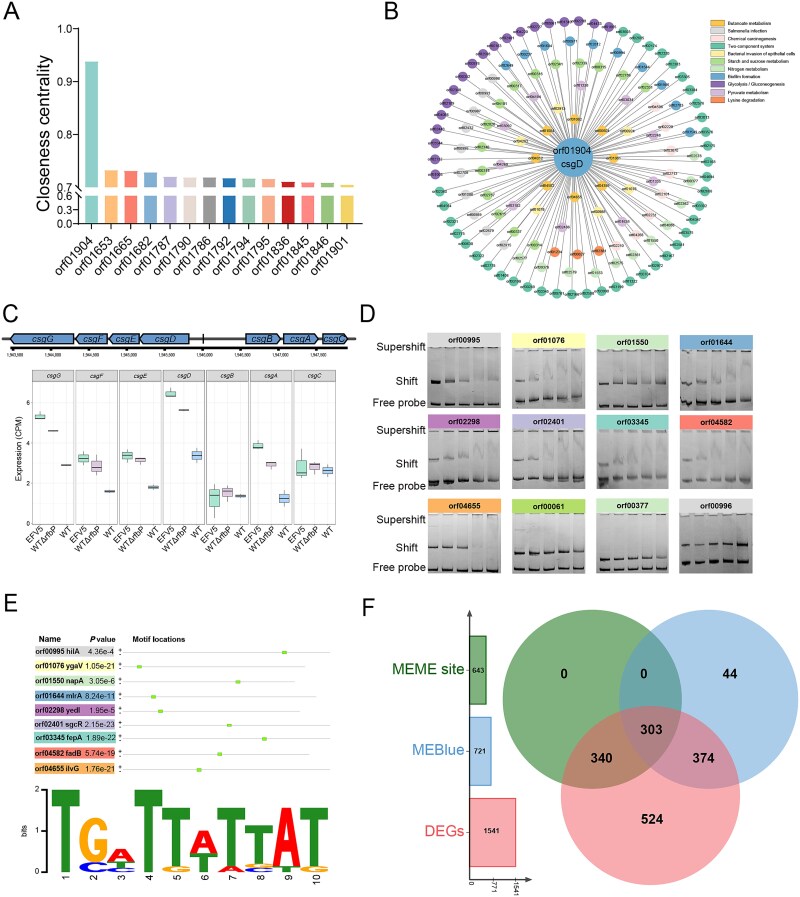
Identification and functional analysis of *csgD*-centered gene regulatory network in the MEblue module. (A) Closeness centrality analysis of genes within the MEblue module. Genes are ranked based on their closeness centrality values, with *csgD* exhibiting the highest centrality, indicating its central role within the MEblue module. (B) Co-expression network centered on *csgD*. Nodes represent genes within the MEblue module that show significant correlation with *csgD*, categorized by different KEGG pathways. The size of the nodes and connections indicates the strength of their association based on Pearson correlation coefficients. (C) Expression profiles of genes in the *csg* cluster based on Counts Per Million (CPM) in WT, EFV5, and WTΔ*rfbP*::*rfbP*_L27*_ strains. The box plots show the expression distribution of each gene in the *csg* cluster, highlighting variations across different strains. (D) Electrophoretic mobility shift assay (EMSA) for the genes correlated with *csgD*. Bands indicate binding interactions of *csgD* with the promoter regions of selected genes. Supershift indicates the presence of *csgD*-DNA complexes, while free probe represents unbound DNA. (E) The predicted *csgD*-binding motif identified through MEME Suite. The logo represents the consensus sequence for *csgD* binding, and a list of genes with the predicted motif occurrences are shown alongside their *P* values. (F) Venn diagram showing the intersection of genes from three categories: MEME site prediction, MEblue module (correlated with *csgD*), and DEGs between EFV5 and WT. The overlap represents genes potentially regulated by *csgD*. The list of genes predicted to be regulated by *csgD* is shown in Table S6.

Detailed analysis of the MEblue module identified 1186 genes. By calculating the closeness centrality of the MEblue module, we found that *csgD* performed the best with a value of 0.9384 (Fig. 6A). Then, we constructed a co-expression network analysis centered on *csgD* using Pearson correlation coefficients to analyze the gene network. Among the 1186 genes from MEblue module, 742 were found to be correlated with *csgD* and enriched in 11 pathways (Fig. 6B). Based on the transcriptome data from the WT, EFV5, and WTΔ*rfbP*, except for *csgB* and *csgC*, the counts per million (CPM) values of other genes in the *csg* cluster were higher in EFV5 and WTΔ*rfbP*, compared to the WT strain, with the *csgD* exhibiting the highest CPM value (Fig. 6C). We selected nine genes representing different pathways in the MEblue module, which are predicted to be correlated with *csgD*, for EMSA. The genes predicted with no correlation with *csgD*—including orf00061, orf00377, and orf00996—were used as controls. EMSA analysis confirmed that CsgD binds to the promoter regions of all nine selected genes, including orf00995, orf01076, orf01550, orf01644, orf02298, orf02401, orf03345, orf04582, and orf04655 (Fig. 6D). The predicted motif for the CsgD-binding promoter on the Multiple Em for Motif Elicitation (MEME) Suite [50] is as follows: TSHTT(K)WT(W)T(B)A(W)T(K) (Fig. 6E). Using the predicted MEME site, we traced the *Salmonella* genome to search for the promoter sequences located 500 bp upstream of genes. A total of 1853 promoters containing this sequence were identified. Furthermore, we intersect them with the DEGs between the WT and EFV5 and the genes within the MEblue module, and identified 303 genes potentially bound by CsgD, which are associated with bacterial motility, biofilm formation, adhesion, invasion, and metabolism (Fig. 6F). Our data imply a wide range of regulatory roles of *csgD* in physiological processes. However, it has to be pointed out that these findings based on EMSA and MEME site prediction require further validation by the use of other techniques, such as DNA footprinting, luciferase reporter assay, or chromatin immunoprecipitation.

### Phenotypic alteration caused by *rfbP* deficiency mostly depends on *csgD* upregulation

**Figure 7:**
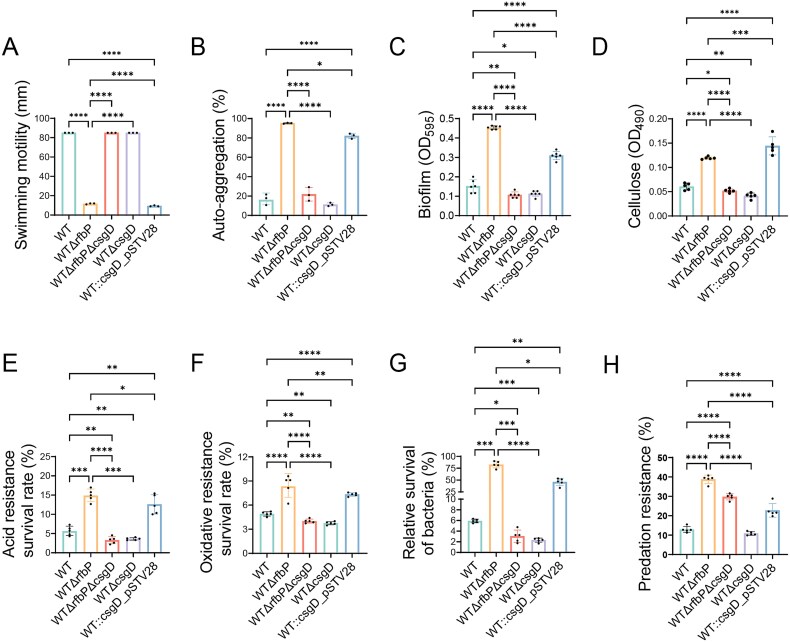
Impact of *csgD* knockout or overexpression on phenotypic traits in WT and WTΔ*rfbP* strains. (A) Bacterial swimming motility on LB soft agar plates. (B) Bacterial auto-aggregation ability. (C) Bacterial biofilm formation ability. (D) Cellulose production of *Salmonella* strains. (E) Acid resistance of *Salmonella* strains. (F) Oxidative stress resistance of *Salmonella* strains. (G) Predation resistance of *Salmonella* strains against *T. thermophila*. (H) Relative survival of *Salmonella* strains after exposure to *T. thermophila*. Error bars represent the standard deviations (*n* = 5) for all the plots. One-way ANOVA test was used to determine statistical significance between the groups. ^*^P < 0.05, ^**^P < 0.01, ^***^P < 0.001, and ^****^P < 0.0001.

To further determine the role of *csgD* in phenotypic alteration caused by inactivation of *rfbP*, we knocked out the *csgD* gene in the background of WTΔ*rfbP*. Deletion of *csgD* almost completely restored the defective motility (Fig. 7A) and decreased the auto-aggregation abilities (Fig. 7B) of WTΔ*rfbP* to the wild-type levels. Compared to the WTΔ*rfbP*, WTΔ*rfbP*Δ*csgD* exhibited substantially decreased levels of biofilm formation (Fig. 7C), cellulose production (Fig. 7D), acid resistance (Fig. 7E), oxidative stress resistance (Fig. 7F), and bacterial survival rates within *Tetrahymena* (Fig. 7G), although these phenotypic changes have not yet returned to the level of the WT strain. Predatory resistance is an exception, since deletion of *csgD* seems not to significantly reduce resistance of WTΔ*rfbP* to phagocytosis by *T. thermophila* (Fig. 7H)*.* We found that deletion of *csgD* in the WT strain did not have a substantial impact on the phenotypes tested in this study, especially with almost no effect on swimming motility, auto-aggregation, and predation resistance; on the contrary, overexpression of *csgD* had an extremely significant effect on the phenotypes of WT, almost consistent with what was observed in the WTΔ*rfbP*. We speculate that these phenotypic changes might be closely related to the transcriptional level of *csgD*. To demonstrate this, we evaluated the *csgD* transcription levels from different bacterial strains (Fig. S2A) and conducted a regression analysis of the relationship between them and various phenotypes tested in this study (Fig. S2B). Our data showed that *csgD* transcription levels were strongly correlated with acid stress tolerance, survival, auto-aggregation, motility, biofilm formation, and cellulose production (*R*^2^ > 0.6) and weakly correlated with oxidative stress resistance and anti-phagocytosis ability (*R*^2^ > 0.3), with the weakest correlation observed for anti-phagocytosis ability (*R*^2^ = 0.34).

### Cyclic diguanylate GMP might play a bridge role between *rfbP* deficiency and *csgD* upregulation

**Figure 8:**
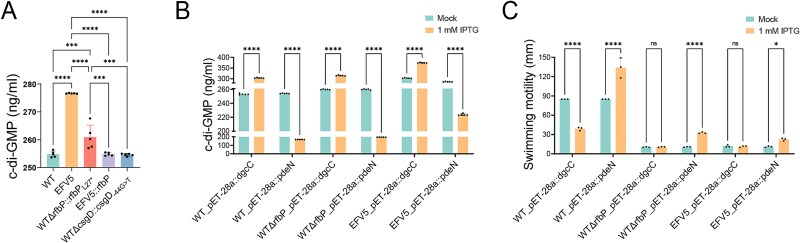
The level of intracellular cyclic di-GMP and its effect on bacterial motility. (A) Intracellular c-di-GMP concentration quantified in the WT parent strain and its derived mutants. Bacterial cultures were grown to logarithmic phase, and the c-di-GMP level (ng/ml) was measured using enzyme-linked immunosorbent assay. Statistical significance was determined using one-way ANOVA. (B) Measurement of intracellular c-di-GMP levels in strains carrying plasmid constructs for diguanylate cyclase (*dgcC*) or phosphodiesterase (*pdeN*). Cultures were treated with 1 mM isopropyl β-D-thiogalactoside (IPTG) to induce expression of the respective enzymes, and c-di-GMP levels were quantified. (C) Swimming motility of bacterial strains after induction of *dgcC* or *pdeN* with IPTG. Bacterial motility was assessed on LB soft agar plates (0.4% agar), and the zone of migration was measured after 12 h of incubation. Statistical analyses (B and C) were performed using two-way ANOVA followed by Sidak’s multiple comparisons test. ^*^P < 0.05, ^**^P < 0.01, ^***^P < 0.001, and ^****^P < 0.0001. “ns” indicates no significant difference.

We sought to determine how the connection between *rfbP* and *csgD* was formed. Among the significantly upregulated genes shared by EFV5 and WTΔ*rfbP*, we identified those that are known to be strongly associated with osmotic pressure protection, including the *osm* gene family (*osmB*, *osmC*, *osmE*, *osmF*, *osmW*, *osmV*, *osmX*, and *osmY*), the *yeh* gene family (*yehW*, *yehX*, *yehY*, and *yehZ*), and metabolic genes for osmoprotectants (*treY*, *treZ*, *treF*, *treA*, *ostA*, and *ostB*) (Fig. S3). This finding led us to test the level of intracellular c-di-GMP, since c-di-GMP has been reported to play vital roles in regulating *csgD* activity [51] and osmolyte transport [52]. As a result, intracellular c-di-GMP levels were markedly increased in WTΔ*rfbP*::*rfbP*_L27*_ and EFV5 when compared with the WT strain, while overexpression of the intact *rfbP* gene in EFV5 restored the c-di-GMP to the WT level (Fig. 8A). To alter the intracellular level of c-di-GMP and investigate its regulatory role in phenotypic traits, we constructed the inducible plasmids containing the *dgcC* (encoding a diguanylate cyclase) [53] or *pdeN* (encoding a phosphodiesterase) [54] and transformed them into the WT, WTΔ*rfbP*, and EFV5 strains. After being induced by 1 mM isopropyl-beta-d- thiogalactopyranoside (IPTG), all the three bacterial strains overexpressed with *dgcC* or *pdeN* exhibited significantly up- or downregulated c-di-GMP levels (Fig. 8B). Further, we examined the bacterial motility, which is used as an example for phenotypic observation. Our data showed a negative relationship between intracellular c-di-GMP level and motility. Overexpression of *pdeN* significantly increased bacterial motility in the above three strains, while the overexpression of *dgcC* markedly reduced bacterial motility in the WT strain, although no further decrease in originally repressed mobility was found in the WTΔ*rfbP* and EFV5 strains (Fig. 8C).

## Discussion

Many pathogenic bacteria live in close association with protozoa [55]. In response to protozoan predation, they have evolved various strategies, e.g. biofilm formation, changes in size and motility, and the production of extracellular matrix, to minimize the risk of being preyed upon [56]. The predation pressure may induce genetic changes in bacteria, maintaining their adaptive mechanisms [4]. In this study, we focus on the EFVs-derived *Salmonella* and explore their phenotypic and genomic evolution. Our results indicated that the former populations (EFV1 − EFV3) after passage through *Tetrahymena* have not exhibited any alterations in all phenotypes tested, but some EFV4 (EFV4-R) and all the EFV5 populations displayed substantial phenotypic changes, including enhanced auto-aggregation, biofilm formation, intracellular survival, oxidative resistance, and acid resistance, but reduced motility. Further evidence for the presence of random mutations in early passages (EFV1 − EFV3)—along with the fixation (100%) of two beneficial mutations in *rfbP* and *csgD* in the EFV4-R and EFV5 populations—strongly suggests adaptive evolution in *Salmonella*. This mutation in the *rfbP* gene generates a stop codon that disrupts protein translation and greatly contributes to the observed phenotypic alterations in the EFV5, whereas another mutation occurring in the *csgD*, although related to phenotype to some extent, has a relatively small contribution.

The *rfbP* gene has been known to be involved in the first step of LPS O-antigen synthesis in *S. enterica* by catalyzing the transfer of galactose 1-phosphate onto undecaprenyl phosphate [46]. Therefore, this mutation within the *rfbP* gene caused an obstruction of O-antigen synthesis and thus changed the structural integrity of LPS in *Salmonella.* LPS plays a critical role in maintaining the outer membrane of Gram-negative bacteria, contributing to bacterial defense mechanisms against host immune responses [57]. We speculate that LPS deficiency represents an adaptive evolution driven by protozoan predation, enabling the bacteria to evade recognition. Consistent with this idea, this mutation in the *rfbP* gene increased the resistance of *Salmonella* to phagocytosis by *T. thermophila* in this study.

Currently, little is known about the functions of *rfbP*, except for involvement in bacterial O-antigen synthesis. Considering the *rfbP* gene mutation resulted in multiple phenotypic changes in *Salmonella*, we aimed to identify additional functional genes potentially contributing to this effect. Therefore, we made a transcriptomic RNA-Seq comparison between WT, EFV5, and WTΔ*rfbP*::*rfbP*_L27*_. Compared to the WT strain, we identified an overlap of 708 DEGs in EFV5 and WTΔ*rfbP*::*rfbP*_L27*_. Most of the shared DEGs are linked to bacterial metabolism, consistent with what was observed in *V. cholerae* [19] and *A. hydrophila* [15], which have adapted to predation pressure from *T. thermophila*. These findings highlight the intricate adaptive responses of bacteria to protozoan predation, reflecting adjustments in internal BPs to enhance environmental tolerance and survivability.

To elucidate how *rfbP* deficiency works in inducing broad transcriptomic changes in *Salmonella*, we performed the WGCNA to pinpoint key genes and identified the MEblue module with *csgD* displaying high centrality. This observation attracted our attention, as *csgD* is associated not only with biofilm formation but also with metabolic growth and other biological functions. *CsgD* has been known to play an important role in regulating biofilm formation and bacterial community behavior [53], but other functions have not yet been reported. Further, based on the prediction of the potential *csgD*-binding site, although still to be confirmed, we obtained 303 possible target genes directly regulated by *csgD* in the EFV5, signifying its regulatory role in a diversity of cell functions. These additional functional consequences in the *csgD* remain an area of our ongoing research. Nevertheless, our study demonstrates that phenotypic alterations in the WTΔ*rfbP* are most likely due to upregulation of *csgD*, as evidenced by the fact that inactivation of *csgD* restored phenotypic changes caused by deletion of the *rfbP*. A regression analysis of the relationship between *csgD* transcription levels and different phenotypes further supports this hypothesis. However, a question is rising as to why a mutation at the -44 position of the *csgD* promoter does not contribute as much to the phenotypic changes of EFV5 as the *rfbp* deletion. We speculate that this is most likely due to the fact that the transcription level of *csgD* caused by this mutation is not as high as that in the WTΔ*rfbP*. Furthermore, it appears that its overexpression of *csgD*, but not its lacking, is associated with the phenotypic traits tested, since lacking of *csgD* does not apparently cause phenotypic alteration in the WT or WTΔ*rfbP* background, except for biofilm formation and cellulose production.

The mechanisms whereby *csgD* is involved in the regulation of phenotypic traits in the WTΔ*rfbP* strain are not clear at this time. However, our transcriptome data indicated that osmolarity-associated genes from different families were significantly upregulated in the WTΔ*rfbP.* Furthermore, we provide evidence that intracellular c-di-GMP levels were markedly elevated in the WTΔ*rfbP*. The c-di-GMP, acting as a secondary messenger, enables bacteria to rapidly respond to environmental change without requiring transcription or translation [58, 59]. It can help maintain cell wall stability through osmolyte regulation [60]. Therefore, it is reasonable to assume that in the absence of *rfbP*, compromised cell wall integrity necessitates a reduction in internal turgor pressure via c-di-GMP-dependent osmolyte transport regulation. In addition, the interplay between c-di-GMP and *csgD* has been well-established [61–63]. Increased c-di-GMP boosts *csgD* expression, which, in turn, provides positive feedback for c-di-GMP production [47]. This creates a cascade amplification effect, resulting in elevated c-di-GMP and enhanced *csgD* expression. We speculate that phenotypic changes in the WTΔ*rfbP* might be associated with the levels of c-di-GMP. Consistent with this idea is the observation that intracellular c-di-GMP levels exhibited the perfect negative correlation with bacterial motility in different bacterial strains. Especially, the originally repressed motility in the WTΔ*rfbP* strain was significantly improved due to reduced intracellular c-di-GMP levels by the overexpression of *pdeN*. This finding indicates that high expression of c-di-GMP is fully or at least partially responsible for the impaired motility of WTΔ*rfbP*. Additionally, overexpression of *dgcC* elevated intracellular c-di-GMP level, but did not significantly affect the motility of WTΔ*rfbP*. A reasonable explanation for this phenomenon is that deletion of *rfbP* almost completely impairs bacterial motility, which makes it impossible to see further reduction in motility even when the c-di-GMP level is largely elevated. Based on the above findings, we hypothesize that c-di-GMP might play a bridge role between *rfbP* deficiency and *csgD* upregulation. While our data support this logical relationship, the exact mechanism by which *rfbP* affects *csgD* transcription requires further investigation.

Despite uncovering significant insights into the role of predation pressure in the adaptive evolution of *Salmonella*, this study has limitations. First, our experiments were conducted under laboratory conditions, which may not fully reflect the complexity of natural environments. Second, our focus was mainly on *csgD* and its regulatory influence on phenotypic traits. Indeed, genes related to bacterial adhesion, invasion, and carbon source utilization were also upregulated in *Tetrahymena*-adapted isolates, e.g. the type III secretion system (T3SS). T3SS is a critical virulence factor in *Salmonella* that enhances bacterial survival and proliferation within host cells [64, 65]. However, we found that the adapted strain EFV5 exhibited significantly reduced virulence in mice (data not shown). This apparent contradiction remains to be elucidated in further study. Additionally, upregulation of genes related to carbon source utilization implies metabolic flexibility, which aids bacteria in adapting to variable nutrient environments [66–69]. Our future work is to investigate the role of carbon source utilization in *Salmonella* survival under predation pressure.

Overall, *rfbP* deficiency and the resulting upregulation of the *csgD* in *Tetrahymena*-adapted *Salmonella* represent an evolutionary trade-off by which bacteria develop some strategies to overcome environmental adversities, including reduced motility and increased biofilm formation. Our findings support the hypothesis that protozoan predation promotes the selection of a more “chronic” state of coexistence. Understanding the complex interactions between bacteria and protozoa can elucidate bacterial adaptive strategies and aid in developing novel targets and strategies for anti-infection therapies.

## Supplementary Material

Fig-S1_wraf070

Fig-S2_wraf070

Fig-S3_wraf070

Supplement_Material_wraf070

Supplementary_information_wraf070

## Data Availability

Sequencing data are available at NCBI under BioProject PRJNA1110004.
