# Supplementary figures and images for: Tetrahymena predation drives adaptive evolution of Salmonella by disrupting O-antigen biosynthesis and upregulating transcriptional regulator csgD
Source: ISME J. 2025 Apr 14;19(1):wraf070. doi: 10.1093/ismejo/wraf070 (PMC12061854; doi:10.1093/ismejo/wraf070)

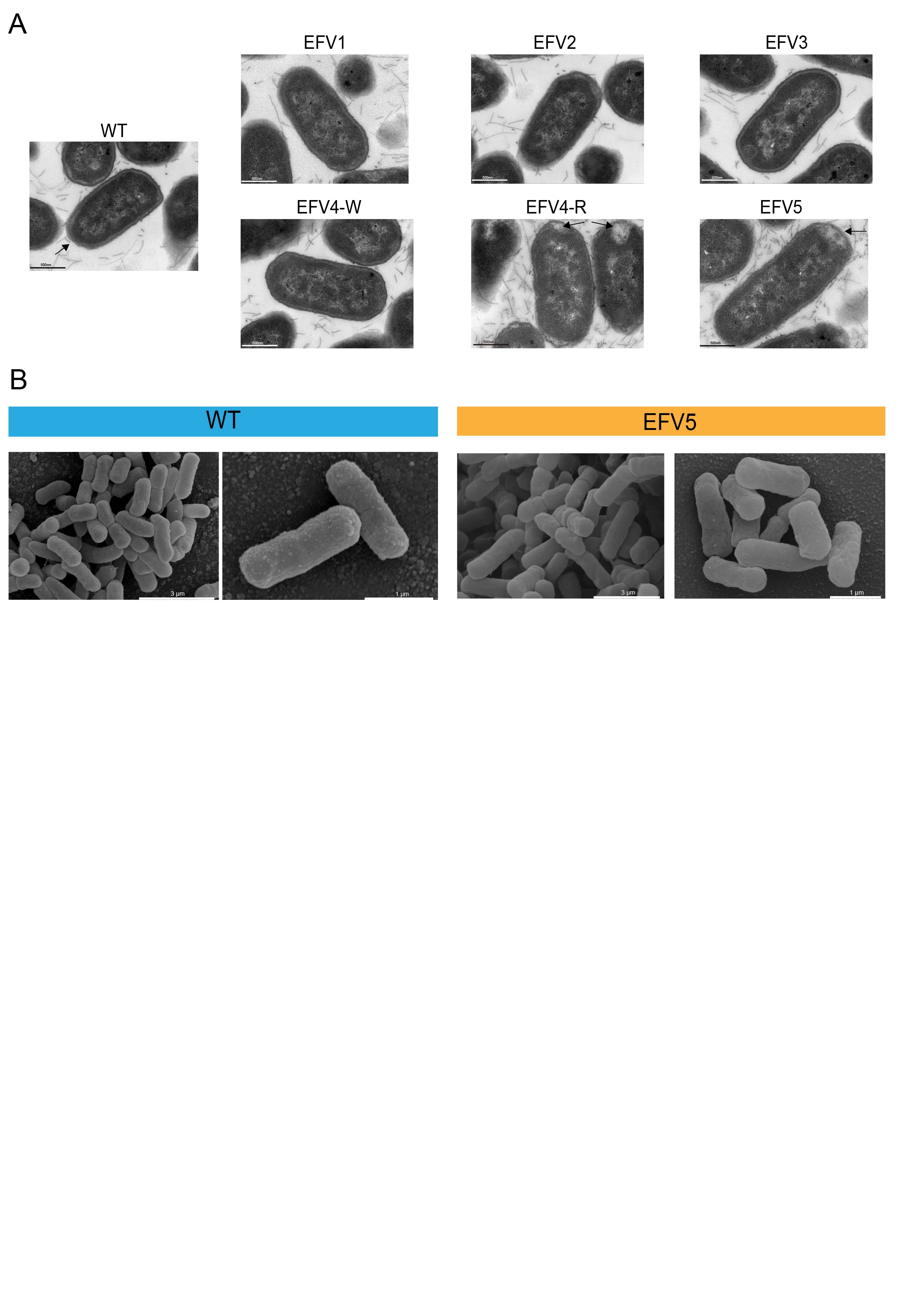

Supplement: Fig-S1_wraf070 [file fig-s1_wraf070.jpeg]

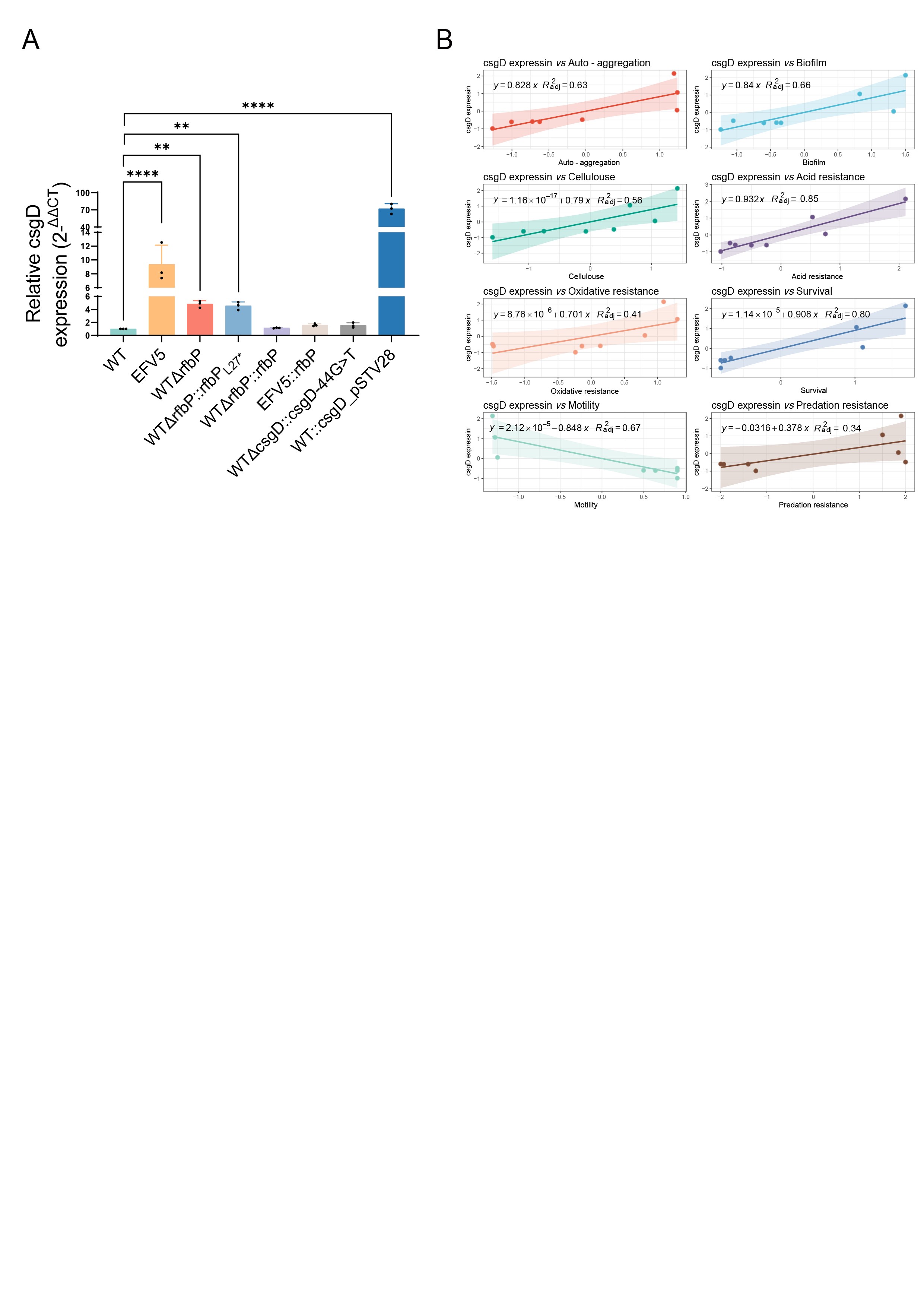

Supplement: Fig-S2_wraf070 [file fig-s2_wraf070.jpeg]

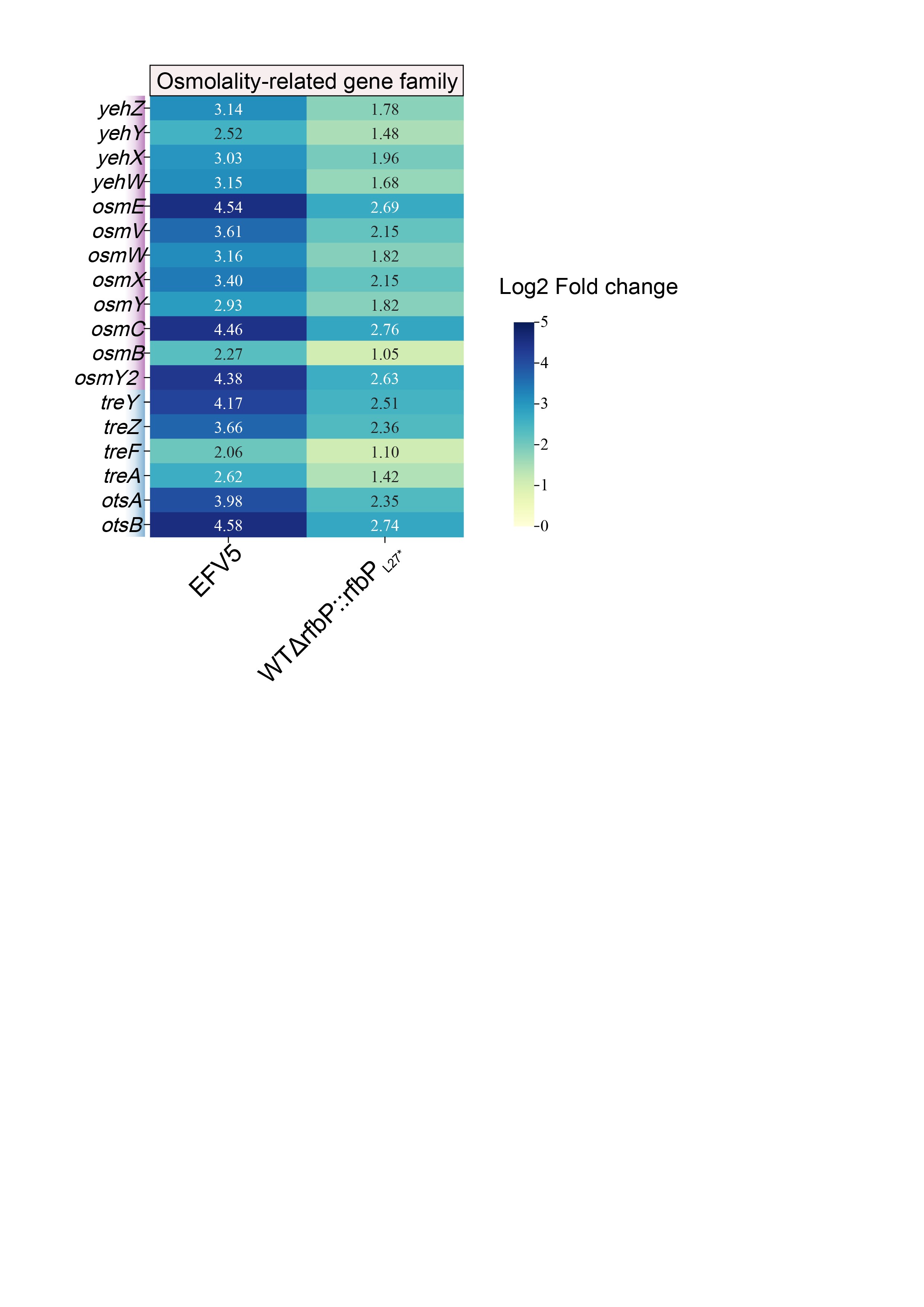

Supplement: Fig-S3_wraf070 [file fig-s3_wraf070.jpeg]
