# Supplementary material for: Tetrahymena predation drives adaptive evolution of Salmonella by disrupting O-antigen biosynthesis and upregulating transcriptional regulator csgD
Source: ISME J. 2025 Apr 14;19(1):wraf070. doi: 10.1093/ismejo/wraf070 (PMC12061854; doi:10.1093/ismejo/wraf070)
Supplement: Supplementary_information_wraf070 [file supplementary_information_wraf070.docx]

**Supplementary information**

**Figure S1.** Microscopic analysis of WT and EFV-adapted strains. (A) Transmission electron microscopy (TEM) images of WT and EFV-adapted *Salmonella* strains. Scale bar: 500 nm. (B) Scanning electron microscopy (SEM) images of WT and EFV5 strains. Scale bar: 1 µm.

**Figure S2.** The *csgD* transcription level and its correlation with phenotypic traits in different bacterial strains. (A) Relative expression of the *csgD* gene (2^−ΔΔ^*^C^*^t^) in different bacterial strains, including WT, EFV5, WTΔ*rfbP*, WTΔ*rfbP*::*rfbP*_L27*_, and strains with *csgD* knockout or overexpression. The bars indicate the average expression levels of *csgD* with standard deviation

across biological replicates. (B) Regression analysis of the *csgD* expression level and bacterial phenotypic traits, including auto-aggregation, biofilm formation, cellulose production, acid resistance, oxidative stress resistance, survival, motility, and predation resistance. The scatter plots show linear relationships between *csgD* expression and each phenotype, with the corresponding regression line, *R*² values, and trend equations. Each point represents a strain, and the shaded area indicates the 95% confidence interval of the regression line.

**Figure S3.** Expression analysis of osmolality-related genes in *Salmonella* strains. Heatmap of expression levels of osmolality-related genes across different strains, including the *osm* gene family, *yeh* gene family, and other osmotically inducible genes. The color gradient represents log2 fold changes of gene expression.
